# Energy metabolic pathways in neuronal development and function

**DOI:** 10.1093/oons/kvad004

**Published:** 2023-03-21

**Authors:** Sebastian Rumpf, Neeraja Sanal, Marco Marzano

**Affiliations:** Multiscale Imaging Center, University of Münster, Röntgenstrasse 16, 48149 Münster, Germany; Multiscale Imaging Center, University of Münster, Röntgenstrasse 16, 48149 Münster, Germany

**Keywords:** synapse, dendrite, axon, neuron, oxidative phosphorylation, glycolysis

## Abstract

Neuronal development and function are known to be among the most energy-demanding functions of the body. Constant energetic support is therefore crucial at all stages of a neuron’s life. The two main adenosine triphosphate (ATP)-producing pathways in cells are glycolysis and oxidative phosphorylation. Glycolysis has a relatively low yield but provides fast ATP and enables the metabolic versatility needed in dividing neuronal stem cells. Oxidative phosphorylation, on the other hand, is highly efficient and therefore thought to provide most or all ATP in differentiated neurons. However, it has recently become clear that due to their distinct properties, both pathways are required to fully satisfy neuronal energy demands during development and function. Here, we provide an overview of how glycolysis and oxidative phosphorylation are used in neurons during development and function.

## MAIN

The nervous system is built to perform highly specific and fast computations. These functions constantly demand a high amount of energy in the form of adenosine triphosphate (ATP)—in fact, in humans at rest, the brain, 2.5% of total body weight, uses 20% of the body’s energy. The construction of precisely connected neural circuits in the brain during development is equally energy-demanding, and brain energy demand is even higher in young children, when circuits are fine-tuned by synapse formation and pruning [1]. The ATP-providing pathways, glycolysis and the tricarboxylic acid (TCA) cycle followed by oxidative phosphorylation, are therefore highly active in the nervous system. These two pathways are linked because pyruvate, the product of glycolysis, is a starting point for the TCA cycle, but they have distinct properties regarding their efficiency and compatibility with other metabolic pathways, such that their balance must be constantly adapted to the specific purpose. Here, we provide an overview over the regulation and differential use of these two pathways in the nervous system during development and function.

## GLYCOLYSIS VERSUS THE TCA CYCLE

**Figure 1:**
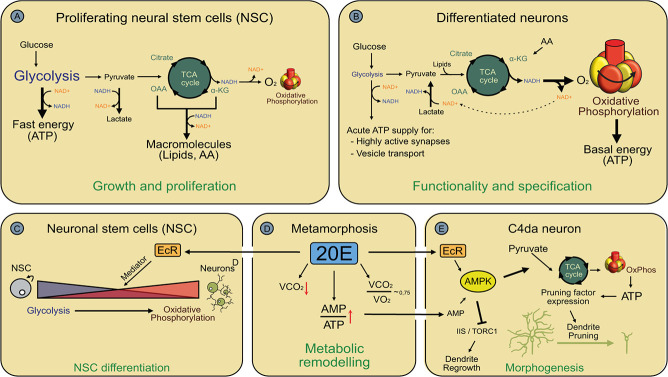
Use of glycolysis, the TCA cycle and oxidative phosphorylation during neuronal development. **A,** Use of glycolysis, the TCA cycle and oxidative phosphorylation in neuronal stem cells. AA, amino acids. **B,** Use of glycolysis, the TCA cycle and oxidative phosphorylation in differentiated neurons. **C,** Regulation of glycolysis and oxidative phosphorylation during neuronal differentiation in *Drosophila*. **D,** The systemic metabolic switch at the onset of the pupal stage in *Drosophila*. The VCO_2_/VO_2_ ratio enables conclusions about metabolic fuel usage. **E,** Regulation of energy metabolism during neurite pruning, likely in response to the metabolic switch depicted in (D). 20E, 20-hydroxy-ecdysone; VCO_2_, CO_2_ release as measure of metabolic rate; VO_2_, oxygen consumption.

Glycolysis encompasses 10 enzymatic reactions in the cytoplasm that break down glucose to two molecules of pyruvate. These reactions yield two molecules of ATP and reduction equivalents in the form of two NADH molecules. If demanded by the cellular redox balance, pyruvate can be reduced to lactate by using the NADH molecules generated during the early glycolytic reactions (Figure 1 A, B). Otherwise, pyruvate is imported into mitochondria for use in the TCA cycle, where it is decarboxylated to acetyl-CoA. It is then fused to oxaloacetate to generate citrate. In a series of eight enzymatic reactions, the acetate moiety is oxidized to yield reduction equivalents (NADH and FADH_2_) and two molecules of CO_2_, whereas oxaloacetate is recycled for further rounds of pyruvate oxidation. NADH and FADH_2_ are oxidized to NAD^+^ and FAD in the electron transport chain at the inner mitochondrial membrane as the electrons are transferred to molecular oxygen to yield water. The energy provided by these reactions is used to generate the proton gradient that powers ATP synthase. In this way, the oxidation of one molecule of glucose yields 32 molecules of ATP.

Oxidative phosphorylation is much more efficient than glycolysis, so many cell types prioritize it as long as oxygen is present. The TCA cycle also allows for metabolic flexibility because it can run on other substrates than glucose. For example, pyruvate and the cycle intermediates fumarate and alpha-ketoglutarate can be generated by deamination of amino acids, and acetyl-CoA can be produced from fatty acid breakdown. On the other hand, TCA cycle intermediates serve as building blocks for anabolic pathways such as amino acid and nucleotide synthesis as well as gluconeogenesis. Thus, not all pyruvate channeled into the TCA cycle may be used for ATP production. Because of the cyclic nature of the TCA cycle, intermediates can only be taken out—through so-called cataplerotic reactions—if they are replenished through a so-called anaplerotic reaction, or the pathway will run dry (Figure 1 B). The presence of oxygen, a key prerequisite for oxidative phosphorylation, often suppresses glycolysis, a phenomenon known as the ‘Pasteur effect’ [2]. Mature neurons in the well-vascularized brain are therefore thought to rely heavily on oxidative phosphorylation, and it has been proposed that they—to some degree—‘outsource’ glycolysis to glial cells and burn lactate or amino acids derived from them [3].

## METABOLIC REGULATION DURING NEURONAL STEM CELL DIVISION

Neurons are terminally differentiated postmitotic cells. They are generated from neuronal stem cells (NSCs) that first expand through symmetric cell divisions and then divide asymmetrically to give rise to differentiating neurons. Once the number of differentiated neurons approaches the total number of neurons in a given nervous system, precursors stop divisions and undergo differentiation themselves.

Many types of stem cells, including NSCs, have an active glycolytic pathway for ATP generation, possibly in part because stem cells try to avoid damage from reactive oxygen species (ROS) generated in the electron transport chain [4, 5]. Even though NSCs in diverse models are located in less oxygenated environments [6, 7], oxygen is likely present in these cells because oxidative ATP production is still important. This phenomenon of aerobic glycolysis is called the ‘Warburg effect’. Use of glycolysis therefore likely also enables NSCs to use TCA cycle intermediates for anabolic purposes such as amino acid and nucleotide biosynthesis. With this important role, active regulation of glycolysis and TCA cycle/oxidative phosphorylation contributes to the balance between cell division and differentiation. *Drosophila melanogaster* NSCs, also known as neuroblasts, undergo asymmetric cell divisions to generate a smaller cell—or a limited lineage of smaller cells—committed to neuronal differentiation, and a larger undifferentiated neuroblast that retains its proliferative potential. *Drosophila* is a holometabolic insect and the adult brain is built during metamorphosis. During early metamorphosis, neuroblasts cease cell divisions, become smaller and eventually undergo a terminal symmetric division that gives rise to two differentiating daughter cells [8]. The end of the proliferative phase at the onset of the pupal stage is induced by a transcriptional programme that is governed by the molting hormone 20-hydroxy-ecdysone (also known as 20E, or ecdysone). Ecdysone is a steroid hormone that acts through its corresponding steroid hormone receptor EcR together with Mediator, a transcriptional coactivator complex [9]. A significant part of this transcriptional programme affects metabolic genes, with downregulation of glycolytic enzymes and concomitant upregulation of oxidative phosphorylation, leading to a corresponding switch in energy metabolism (Figure 1 C). Importantly, partial inhibition of oxidative phosphorylation in early pupal neuroblasts prevents the switch to differentiation and causes neuroblasts to continue cell divisions, indicating that the metabolic programme is partially causative of the differentiation switch [9]. However, the TCA cycle and oxidative phosphorylation must also be active in NSCs because stronger inhibition of these pathways leads to early defects in the timing of cell divisions, thus likely affecting the later identity of the newborn neurons [10].

Genetic studies on mammalian NSCs, and in particular human evolution, support the observation that metabolic regulation plays an important role in supporting NSC divisions during brain development. For example, mice lacking the enzyme Arginase-II display altered localization and lower activity of the glycolytic enzyme hexokinase in adult NSCs. These NSCs produce ATP predominantly through oxidative phosphorylation, leading to premature differentiation and a lower rate of adult neurogenesis [11]. Compared with other mammals, the human cortex is characterized by a very high number of neurons. This is achieved by an extended phase of cell divisions of the corresponding cortical NSCs, the so-called basal radial glia (bRG). Research into the mechanisms underlying the extraordinarily high proliferative capacity of this cell type in humans identified ARHGAP11B, a gene that arose after the evolutionary split between humans and chimpanzees through duplication and is highly expressed in bRGs [12]. ARHGAP11B protein localizes to mitochondria and alters mitochondrial physiology to enhance glutaminolysis, an anaplerotic reaction that channels alpha-ketoglutarate into the TCA cycle [13]. This can either serve to burn amino acids for ATP generation, or to replenish the TCA cycle when intermediates are taken out at other stages, e.g., for nucleotide biosynthesis. Transgenic expression of ARHGAP11B in mouse bRGs led to higher precursor numbers and partial cortical folding [12], indicating that improved supply of energy and anabolic building blocks increases proliferative capacity of neural precursor cells.

Thus, many lines of evidence suggest that neuronal stem cells use glycolysis for ATP production to protect from ROS-induced damage and to enable the use of TCA cycle intermediates for anabolic purposes. A switch from glycolysis to oxidative phosphorylation promotes differentiation.

## METABOLIC REGULATION DURING NEURONAL DIFFERENTIATION AND MORPHOGENESIS

Neuronal differentiation involves the growth and determination of axons and dendrites, synapse formation and specification of the resulting neural network by neurite and synapse pruning and regrowth.

In dissociated murine cortical neurons, both glycolysis and mitochondrial ATP-generating pathways are strongly upregulated during differentiation [14]. Both inhibition of glycolysis and glutamate production—potentially as alternative TCA cycle fuel—decrease neurite growth [14]. Similarly, inhibition of astrocytic lactate production or of neuronal lactate transport (in the MCT2 lactate transporter knockout) led to shorter axons in cultured cortical neurons [15]. Inhibition of energy production leads to strong defects in both axonal and dendritic morphogenesis in many different systems. For example, the localization of mitochondria at branch points is required for normal axonal arborization of murine cortical neurons both *in vivo* and *in vitro* [16] as well as in chicken sensory neurons, where they might support translation [17]. In *Drosophila* sensory neurons, a mutation that alters mitochondrial morphology and function strongly reduces dendrite growth and complexity [18, 19]. In the same system, a mutation in the energy homeostasis regulator AMP-activated protein kinase (AMPK, see also further) also causes a strong reduction in dendrite growth and complexity with abnormal dendritic actin accumulations [20]. Building blocks for anabolic processes, like acetyl-CoA, are also likely continuously needed for growth of large dendritic arbors. For example, regulators of lipid synthesis are required for both dendrite growth and neuronal function in *Drosophila* sensory neurons [21, 22].

Glycolysis is also important for neuronal morphogenesis. Glycolytic enzymes are localized to axonal growth cones, and glycolysis inhibition causes rapid growth cone collapse and subsequent stalling of axon outgrowth due to ATP depletion *in vitro* [23]. Interestingly, growth cone collapse was accompanied by abnormal accumulation of actin filaments [23], suggesting that actin dynamics may be the major local ATP-consuming pathway in growth cones, consistent with previous observations [24]. Why is glycolysis important for neurite formation, even though mitochondria are readily transported into growing neurites? One potential reason is that diffusion of ATP in the cytosol is relatively slow [25], such that high ATP consumption could lead to local ATP depletion in neurite regions inaccessible to mitochondria, e.g., in the actin cortex underneath the plasma membrane, or in small synapses. Glycolytic enzymes are likely more mobile than mitochondria, and could therefore provide ATP in a more localized fashion. In fact, a meta-analysis of gene expression and energy consumption shows a strong correlation between aerobic glycolysis and neuronal development [26].

In response to injury, neurites can activate (re)growth programmes that share many features with developmental neurite growth. All of these programmes must be supported metabolically. During regeneration of injured axons in *Caenorhabditis elegans*, mitochondria localize to the newly formed growth cone for energetic support of regeneration [27], and in mice, enhancing axonal mitochondrial motility by deletion of Syntaphilin, an axonal mitochondrial-tethering factor, improves regeneration outcome [28, 29]. Expression of syntaphilin is is low in developing neurons and only increases upon maturation, suggesting that high mitochondrial mobility may be required during development [30]. PTEN/SOCS3 double-mutant neurons, which show enhanced regeneration in a retinal ganglion cell (RGC) injury model, overexpress the mitochondrial protein Armcx1 [31]. Armcx1 increases the fraction of motile mitochondria in axons when overexpressed and can enhance both normal axon outgrowth in cultured cortical neurons and regeneration of RGC axons [31].

Not only growth processes during neuronal morphogenesis require ATP, but also regulated degenerative processes like neurite pruning. The nociceptive sensory class IV dendritic arborization (c4da) neurons of *Drosophila* larvae undergo large-scale pruning of their long and branched dendrites at the onset of metamorphosis in response to the hormone ecdysone, and the aforementioned energy homeostasis regulator AMPK is required for this process [32, 33] (Figure 1 E). AMPK is a trimeric protein kinase that senses cellular energy status through binding sites for ATP and its breakdown products adenosine monophosphate (AMP) and adenosine diphosphate (ADP). When the AMP/ATP ratio is high, AMPK is activated and downregulates energy-consuming pathways, while at the same time stimulating energy production [34]. The involvement of AMPK seems to be due in part to a systemic metabolic switch that occurs in response to ecdysone during the larval-to-pupal transition [35] (Figure 1 D). Metabolic rates decrease dramatically during this period [36], and glucose usage is suppressed to be able to energetically support later events during metamorphosis [35]. As a consequence, early *Drosophila* pupae preferentially burn amino acids and lipids instead of glucose during this stage [35, 36]. This metabolic switch leads to a starvation-like state and increases the AMP/ATP ratio [37], consequently activating AMPK also in c4da neurons [32]. To produce ATP efficiently from other fuels than glucose, AMPK promotes oxidative phosphorylation over glycolysis as well as increased pyruvate channeling into the TCA cycle, likely in part through the anaplerotic enzyme pyruvate carboxylase [32]. A major source of amino acids in cells is proteasomal protein degradation [38], and AMPK also promotes proteasome activity in c4da neurons [32, 33]. In support of the notion that AMPK activation allows neuronal metabolism to burn amino acids, the pruning process becomes highly sensitive to amino acid starvation in the absence of AMPK [32]. C4da neuron dendrite pruning involves a neuronal transcriptional programme that leads to expression of cytoskeletal regulators, thus promoting local neurite degeneration [39, 40]. Loss of AMPK and amino acid starvation prevents expression of the large (4723 amino acids) actin-severing enzyme Mical, indicating that gene expression is particularly affected by a reduction in ATP, likely at the stage of translation [32]. AMPK also counteracts the Target of rapamycin Complex 1 (TORC1) kinase during c4da neuron dendrite pruning, a known activator of mRNA translation [33]. It is interesting to speculate that TORC1 inhibition saves energy, but negative regulation of TORC1 is likely also a regulatory mechanism during development because it is reactivated after pruning to promote regrowth of adult-adapted sensory dendrites [41]. TORC1 inhibition is also required for macroautophagy during postsynaptic pruning in mouse cortical neurons [42], suggesting at least partial conservation of metabolic control during pruning.

**Figure 2:**
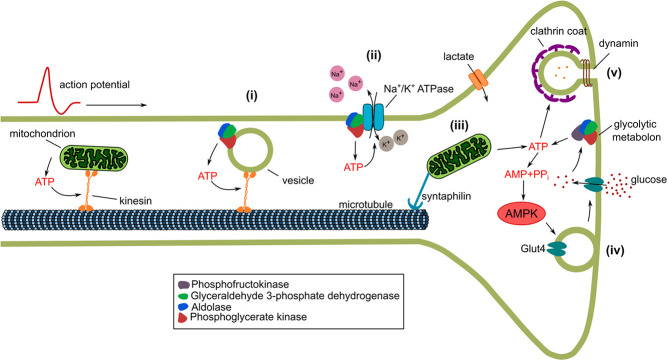
Functions of glycolysis and mitochondrial ATP generation during synaptic function. Energy-demanding processes depicted are (i) glycolysis-powered vesicle transport along microtubules, (ii) energy supply for the Na^+^/K^+^ ATPase by glycolysis, (iii) tethering of mitochondria at synapses, (iv) Glut4 exocytosis at active synapses and (v) endocytic vesicle blocked on ATP depletion.

Taken together, both neurite growth and pruning are energy-demanding, and neurons use both glycolysis (for fast local ATP production) and oxidative phosphorylation (e.g., to adapt to organismal metabolism) to support these morphogenetic events.

## ATP PRODUCTION IN MATURE NEURONS: FAST AXONAL TRANSPORT AND SYNAPTIC FUNCTION

The function of mature neurons is to receive and transmit information through conduction of action potentials along axons and synaptic transmission. These processes require the transport of synaptic components as well as hormones and growth factors along axons, the maintenance of the plasma membrane potential and gradients of key ions and fast neurotransmitter release and recycling. All of these functions require energy that must be continuously provided. One way of providing ATP to axons and synapses is through transport of mitochondria to synapses along axonal microtubules [43] (Figure 2). Synaptic function in particular requires ATP, and there are several mechanisms that link mitochondrial transport to neuronal activity. Mitochondrial transport is locally inhibited by Ca^2+^, which increases at synapses when action potentials arrive. One target of Ca^2+^-mediated inhibition is the mitochondrial kinesin adaptor Miro [44–46]. In addition to the modulation of transport adaptors, mitochondria can also be localized in the vicinity of synapses through specific tethers like Syntaphilin [30], and additional actin-based tethering mechanisms may exist [47]. Mitochondria localized at synapses can remain stationary for long times and cannot be easily mobilized [47]. Mitochondrial tethering can be enhanced in an AMPK-dependent manner, thus directly linking tethering to local ATP depletion [48].

Microtubule-based transport itself is energy-dependent, and mitochondria can provide the ATP for their own transport, but how is this achieved for axonal vesicles carrying growth factors or synaptic components? It turns out that such axonal vesicles are associated with glycolytic enzymes [49], and the complement of glycolytic enzymes found in vesicle fractions supports ATP generation from glucose [50]. In support of the idea that enzyme association with vesicles serves to provide ATP for fast transport, knockdown of glycolytic enzymes in neuronal cultures or *Drosophila* motoneurons selectively reduces the velocity of axonal vesicles, but not mitochondria. Conversely, knockdown of the axonal mitochondrial kinesin adaptor Miro does not affect vesicle speed [49].

An important energy-consuming pathway in resting neurons is likely the maintenance of the membrane potential, which is governed by the Na^+^/K^+^ ATPase. Glycolytic enzymes are found in membrane fractions from many cell types, and they also copurify biochemically with many crucial transporters, including the Na^+^/K^+^ ATPase [51]. Restoration of membrane potential after an action potential is predicted to be one of the most energy-demanding processes in the brain [52]. At the Calyx of Held, the action potential wave form depends on glycolysis in a way consistent with delayed repolarization [53]. In support of a regulatory link between glycolysis and membrane potential control, recent evidence suggests that the Na^+^/K^+^ ATPase directly regulates glycolytic flux [54]. Thus, the Na^+^/K^+^ ATPase could promote local glycolytic ATP production to ensure immediate axonal membrane repolarization after neuronal activity (Figure 2).

Modeling suggests that release of one glutamate neurotransmitter vesicle might cost as much as 20 000 ATP molecules [52]. Sensor measurements in cultured hippocampal neurons suggest that a typical synaptic bouton contains 1 × 10^6^ ATP molecules [55], which could potentially be rapidly depleted on enhanced activity. Indeed, high-frequency stimulation in the presence of inhibitors of glycolysis and oxidative phosphorylation quickly depletes ATP. ATP depletion could be prevented by inhibition of vesicle recycling, suggesting that the most energy-demanding step is before or at the step of dynamin-dependent endocytosis [55]. Similarly, ATP depletion by inhibition of glycolysis and oxidative phosphorylation caused a block in compensatory endocytosis [56]. Loss of presynaptic ATP supply can also lead to increased frequency of mini-excitatory postsynaptic potentials (mEPSPs), indicative of uncontrolled vesicle release [57]. Conversely, neuronal activity stimulates ATP synthesis, suggesting tight coupling between energy usage and supply [55].

Presynapses are small structures that cannot always accommodate mitochondria [58], but the mitochondrial localization mechanisms mentioned previously often ensure sufficient ATP supply. Whereas inhibition of both oxidative phosphorylation and glycolysis is required to abolish vesicle release in hippocampal neurons [55, 57], mitochondrially derived ATP seems strictly required for recovery of presynaptic function and extended activity [58]. In other systems, loss of glycolysis cannot be compensated. In *C. elegans* neurons, several glycolytic enzymes such as phosphofructokinase localize to presynapses under starvation conditions, and loss of glycolytic function leads to mislocalization of presynaptic components and behavioral defects [58]. Interestingly, the glycolytic enzymes seem to cluster under these conditions to form a ‘glycolytic metabolon’ [59] (Figure 2). The mechanism of this clustering may be similar to phase separation [60]. Glycolysis requires glucose as substrate. Mammalian neurons express several glucose transporters, including GLUT3, which can be increased in response to glutamate levels [61, 62], but also GLUT4 [63]. GLUT4 in particular localizes to presynapses where it is exposed at the plasma membrane in an activity-dependent manner [63] (Figure 2). GLUT4 exposure at synapses involves AMPK-dependent phosphorylation of the Rab GTPase-activating protein (RabGAP) TBC1D1 [62], again linking GLUT4 exposure to the AMP/ATP ratio. Functional tests show that GLUT4 is required in hippocampal neurons for memory formation [64].

In conclusion, these studies show that even mature neurons as differentiated cells rely on both oxidative phosphorylation and glycolysis for ATP production. In these cells, the parallel use of these pathways seems to ensure a combination of efficiency oxidative phosphorylation (Oxphos), speed and local supply (glycolysis).

## SUMMARY AND OUTLOOK

Despite the fact that neurons as differentiated postmitotic cells strongly rely on oxidative phosphorylation for ATP production, they also make use of the specific characteristics of glycolysis when needed. In particular, glycolysis is used in neural stem cells, when ROS might be toxic, and in mature neurons, where it can provide ATP more quickly and with higher spatial accuracy, e.g., in small structures like synapses. An emerging theme seems to be the highly localized action of glycolytic enzymes, and future studies may focus on how this spatial specificity can arise. Preferential use of one of the two pathways may also differ between neuronal cell type and model organism, thus, investigation of other models will likely reveal yet other mechanisms for neuronal energy supply. Most of the studies on differential roles of glycolysis and oxidative phosphorylation at synapses discussed here focus on presynaptic mechanisms. Bioenergetics of postsynaptic functions have been discussed elsewhere [65], and it will be interesting to see if postsynaptic energy demands are also met by similarly sophisticated mechanisms. In addition, most studies on the metabolic needs of neurite regeneration focus on mitochondrial transport and placement [27–30]. Because glycolysis is required for actin assembly in growth cones [23], it will be interesting to see whether boosting glycolysis can also enhance regenerative outcomes. Last but not least, it is well established that many neurodegenerative diseases are caused by local energy shortage in axons, and mitochondrial transport defects are thought to underlie many of these [66]. Glycolysis defects have recently been linked to ‘dying back’ pathology under diabetic conditions [67]. Thus, it will be interesting to investigate the interplay between oxidative phosphorylation and glycolysis in neurodegeneration.

## Supplementary Material

Web_Material_kvad004
