# Supplementary material for: Energy metabolic pathways in neuronal development and function
Source: Oxf Open Neurosci. 2023 Mar 21;2:kvad004. doi: 10.1093/oons/kvad004 (PMC10913822; doi:10.1093/oons/kvad004)
Supplement: Web_Material_kvad004 [file Web_Material_kvad004.zip › Reviewers comments - Supplementary Material - OXFNSC-2022-020.R1 .docx]

**REVIEWER COMMENTS**

**OXFNSC-2022-020.R1 - "Energy metabolic pathways in neuronal development and function"**

**Original submission - OXFNSC-2022-020**

**Reviewer 1**

Comments to the Author

This manuscript summarizes the recent studies on energy metabolic pathways in neuronal development and morphogenesis. It is well known that neurons require a large amount of ATPs for their development and function. Two main ATP-producing pathways, glycolysis and oxidative phosphorylation are important for both neuronal stem cells and post-mitotic neurons. This manuscript summarizes the recent progress on metabolic regulation in neuronal stem cell maintenance, dendritic growth and remodelling, as well as synaptic function. Overall, this is a well-written and timely review. I do not have any further suggestion. I strongly support its publication after correcting the following minor point.

Minor point:

Figure 1: should label D and E correctly. The middle panel lacks D while the rightest panel should be E.

**Reviewer 2**

Comments to the Author

I consider the text as an interesting review that deals with an important topic in the field of neurobiology as it studies the energy metabolic pathways in neuronal development and function. The authors analyze recent investigations which have focused in the relevance of ATP production by glycolysis and oxidative phosphorylation in the different steps of neuronal differentiation: neural stem cell proliferation, axonal growth and synapse formation in different animal models.

I have few comments about the manuscript,

1-The review is divided in different sections with titles that introduce different topics. I suggest to include a general conclusion at the end of each section in which the authors discuss each topic: Metabolic regulation during neuronal stem cell division, Metabolic regulation during differentiation and morphogenesis, ATP production in axons…., ATP production and synaptic function. It could be relevant to put the last paragraph of the present review under a new title such Conclusions and Future Directions or Perspectives. This paragraph could also include a deeper discussion about the areas of investigation related to the topic that remain unexplored or are not clear such as the differences in ATP production in the diverse neuronal cell types, the subcellular localization and regulation of the bioenergetic sources in developing neurons and regenerating axon.

2-Regarding the Paragraph under the Title Metabolic regulation during neuronal differentiation and morphogenesis, I consider that it is very interesting but is mainly focus in Drosophila, I consider it would be interesting to include more examples in mammals.

3-I suggest revising carefully the text as there are some repetitions and sentences that are not so clear.

Page 6, line 45 “ One potential reason is that the the diffusibility….” Repetition

Page 6, line 38 “Why glycolysis important for neurite formation, even though mitochondria are upregulated during differentiation and are not only fueled by pyruvate, but also glutamate and lactate delivered by astrocytes?” This sentence is too long and confuse, consider re-writting it.

Page 7, line 31: “ The involvement of AMPK seems in part to be due to….” should be “seems to be due in part to….”

Page 9, line 38 “Membrane fractions from many cell types contain glycolytic enzymes, and glycolytic enzymes are……” Avoid repetition of glycolytic enzymes, replace by “these are….or which are….”.

4- Regarding to the figures:

In Figure1, the panel E is missing. It seems that in this figure, panel D should be E, and the central panel is D.

I suggest to include in the legends some definitions that are missing and appear in the figure such as C4da neuron, VCO2, EcR, 20E for the lectors who are not familiar with the Drosophila melanogaster system.

In Figure2, It is convenient to include the symbols i, ii, iv, and v in the schedule.

**Decision letter - OXFNSC-2022-020**

27-Jan-2023

Dear Dr. Rumpf,

Manuscript ID OXFNSC-2022-020 entitled "Energy metabolic pathways in neuronal development and function" which you submitted to the Oxford Open Neuroscience, has been reviewed. The comments of the reviewer(s) are included at the bottom of this letter.

The reviewer(s) have recommended publication, but also suggest some minor revisions to your manuscript. Therefore, I invite you to respond to the reviewer(s)' comments and revise your manuscript.

Please note that this journal operates with transparent peer review. This means that if your submission is accepted for publication, the full peer review history of your article will publish online alongside your article. This includes reviewer comments, editor decision letters, and your author responses.

To revise your manuscript, log into https://mc.manuscriptcentral.com/oxfnsc and enter your Author Centre, where you will find your manuscript title listed under "Manuscripts with Decisions." Under "Actions," click on "Create a Revision." Your manuscript number has been appended to denote a revision.

You may also click the below link to start the revision process (or continue the process if you have already started your revision) for your manuscript. If you use the below link you will not be required to login to ScholarOne Manuscripts.

*** PLEASE NOTE: This is a two-step process. After clicking on the link, you will be directed to a webpage to confirm. ***

https://mc.manuscriptcentral.com/oxfnsc?URL_MASK=5e2541ee0f95453f8f81935cc24fbfd5

You will be unable to make your revisions on the originally submitted version of the manuscript. Instead, revise your manuscript using a word processing program and save it on your computer. Please also highlight the changes to your manuscript within the document by using the track changes mode in MS Word or by using bold or colored text.

Once the revised manuscript is prepared, you can upload it and submit it through your Author Centre.

When submitting your revised manuscript, you will be able to respond to the comments made by the reviewer(s) in the space provided. You can use this space to document any changes you make to the original manuscript. In order to expedite the processing of the revised manuscript, please be as specific as possible in your response to the reviewer(s).

IMPORTANT: Your original files are available to you when you upload your revised manuscript. Please delete any redundant files before completing the submission.

Because we are trying to facilitate timely publication of manuscripts submitted to the Oxford Open Neuroscience, your revised manuscript should be uploaded as soon as possible. If it is not possible for you to submit your revision in a reasonable amount of time, we may have to consider your paper as a new submission.

Once again, thank you for submitting your manuscript to the Oxford Open Neuroscience and I look forward to receiving your revision.

Sincerely,

Dr. Carlos Ibanez

Senior Editor, Oxford Open Neuroscience

carlos.ibanez@pku.edu.cn

Reviewer: 1

Comments to the Author

This manuscript summarizes the recent studies on energy metabolic pathways in neuronal development and morphogenesis. It is well known that neurons require a large amount of ATPs for their development and function. Two main ATP-producing pathways, glycolysis and oxidative phosphorylation are important for both neuronal stem cells and post-mitotic neurons. This manuscript summarizes the recent progress on metabolic regulation in neuronal stem cell maintenance, dendritic growth and remodelling, as well as synaptic function. Overall, this is a well-written and timely review. I do not have any further suggestion. I strongly support its publication after correcting the following minor point.

Minor point:

Figure 1: should label D and E correctly. The middle panel lacks D while the rightest panel should be E.

Reviewer: 2

Comments to the Author

I consider the text as an interesting review that deals with an important topic in the field of neurobiology as it studies the energy metabolic pathways in neuronal development and function. The authors analyze recent investigations which have focused in the relevance of ATP production by glycolysis and oxidative phosphorylation in the different steps of neuronal differentiation: neural stem cell proliferation, axonal growth and synapse formation in different animal models.

I have few comments about the manuscript,

1-The review is divided in different sections with titles that introduce different topics. I suggest to include a general conclusion at the end of each section in which the authors discuss each topic: Metabolic regulation during neuronal stem cell division, Metabolic regulation during differentiation and morphogenesis, ATP production in axons…., ATP production and synaptic function. It could be relevant to put the last paragraph of the present review under a new title such Conclusions and Future Directions or Perspectives. This paragraph could also include a deeper discussion about the areas of investigation related to the topic that remain unexplored or are not clear such as the differences in ATP production in the diverse neuronal cell types, the subcellular localization and regulation of the bioenergetic sources in developing neurons and regenerating axon.

2-Regarding the Paragraph under the Title Metabolic regulation during neuronal differentiation and morphogenesis, I consider that it is very interesting but is mainly focus in Drosophila, I consider it would be interesting to include more examples in mammals.

3-I suggest revising carefully the text as there are some repetitions and sentences that are not so clear.

Page 6, line 45 “ One potential reason is that the the diffusibility….” Repetition

Page 6, line 38 “Why glycolysis important for neurite formation, even though mitochondria are upregulated during differentiation and are not only fueled by pyruvate, but also glutamate and lactate delivered by astrocytes?” This sentence is too long and confuse, consider re-writting it.

Page 7, line 31: “ The involvement of AMPK seems in part to be due to….” should be “seems to be due in part to….”

Page 9, line 38 “Membrane fractions from many cell types contain glycolytic enzymes, and glycolytic enzymes are……” Avoid repetition of glycolytic enzymes, replace by “these are….or which are….”.

4- Regarding to the figures:

In Figure1, the panel E is missing. It seems that in this figure, panel D should be E, and the central panel is D.

I suggest to include in the legends some definitions that are missing and appear in the figure such as C4da neuron, VCO2, EcR, 20E for the lectors who are not familiar with the Drosophila melanogaster system.

In Figure2, It is convenient to include the symbols i, ii, iv, and v in the schedule.

Associate Editor

Comments to the Author:

(There are no comments.)

**Author response**

We would like to thank the reviewers for their helpful and constructive comments. They helped to significantly improve the manuscript.

Comments to the Author

Reviewer 1.

This manuscript summarizes the recent studies on energy metabolic pathways in neuronal development and morphogenesis. It is well known that neurons require a large amount of ATPs for their development and function. Two main ATP-producing pathways, glycolysis and oxidative phosphorylation are important for both neuronal stem cells and post-mitotic neurons. This manuscript summarizes the recent progress on metabolic regulation in neuronal stem cell maintenance, dendritic growth and remodelling, as well as synaptic function. Overall, this is a well-written and timely review. I do not have any further suggestion. I strongly support its publication after correcting the following minor point.

Minor point:

Figure 1: should label D and E correctly. The middle panel lacks D while the rightest panel should be E.

Our reply: Thank you for this comment. We changed the panel labels accordingly.

Reviewer: 2

Comments to the Author

I consider the text as an interesting review that deals with an important topic in the field of neurobiology as it studies the energy metabolic pathways in neuronal development and function. The authors analyze recent investigations which have focused in the relevance of ATP production by glycolysis and oxidative phosphorylation in the different steps of neuronal differentiation: neural stem cell proliferation, axonal growth and synapse formation in different animal models.

I have few comments about the manuscript,

1-The review is divided in different sections with titles that introduce different topics. I suggest to include a general conclusion at the end of each section in which the authors discuss each topic: Metabolic regulation during neuronal stem cell division, Metabolic regulation during differentiation and morphogenesis, ATP production in axons…., ATP production and synaptic function. It could be relevant to put the last paragraph of the present review under a new title such Conclusions and Future Directions or Perspectives. This paragraph could also include a deeper discussion about the areas of investigation related to the topic that remain unexplored or are not clear such as the differences in ATP production in the diverse neuronal cell types, the subcellular localization and regulation of the bioenergetic sources in developing neurons and regenerating axon.

Our reply: Thank you for these comments. We have now added summaries at the end of each paragraph and a "Summary and outlook" section at the end of the review, where we tried to point out emerging themes and potential future areas of research.

2-Regarding the Paragraph under the Title Metabolic regulation during neuronal differentiation and morphogenesis, I consider that it is very interesting but is mainly focus in Drosophila, I consider it would be interesting to include more examples in mammals.

Our reply: Thank you for this comment. We have now added additional examples for the role of energy supply during neurite morphogenesis (focusing mostly on mitochondrial localization and regeneration).

3-I suggest revising carefully the text as there are some repetitions and sentences that are not so clear.

Page 6, line 45 " One potential reason is that the the diffusibility…." Repetition

Page 6, line 38 "Why glycolysis important for neurite formation, even though mitochondria are upregulated during differentiation and are not only fueled by pyruvate, but also glutamate and lactate delivered by astrocytes?" This sentence is too long and confuse, consider re-writting it.

Page 7, line 31: " The involvement of AMPK seems in part to be due to…." should be "seems to be due in part to…."

Page 9, line 38 "Membrane fractions from many cell types contain glycolytic enzymes, and glycolytic enzymes are……" Avoid repetition of glycolytic enzymes, replace by "these are….or which are….".

Our reply: Thank you for this comment. We have now changed the wording accordingly.

4- Regarding to the figures:

In Figure1, the panel E is missing. It seems that in this figure, panel D should be E, and the central panel is D.

I suggest to include in the legends some definitions that are missing and appear in the figure such as C4da neuron, VCO2, EcR, 20E for the lectors who are not familiar with the Drosophila melanogaster system.

In Figure2, It is convenient to include the symbols i, ii, iv, and v in the schedule.

Our reply: Thank you for pointing these out. We have now extended the legend and corrected the labeling mistake for Figure 1. We also added the numbers to the legend for Figure 2.

**Decision letter - OXFNSC-2022-020.R1**

17-Mar-2023

Dear Dr. Rumpf,

It is a pleasure to accept your revised manuscript entitled "Energy metabolic pathways in neuronal development and function" in its current form for publication in the Oxford Open Neuroscience.

Please note that this journal operates with transparent peer review. This means that the full peer review history of your article will publish online alongside your article. This includes reviewer comments, editor decision letters, and your author responses.

Next steps

You will receive an email from no-reply@scipris.com within roughly one week. This is your invitation to sign up for an account with SciPris, Oxford University Press’ author portal hosted by Aptara. You will need to create an account if you do not already hold one. Please register or log into your account and follow the online instructions which will guide you through signing your licence and paying the APC. The email and the portal have clearly signposted support options if you need any help during this process.

Please note that SciPris is a completely different system from ScholarOne, so your credentials to submit your manuscript here will not work there. Once you’ve created a SciPris account, you will be able to use it whenever you publish with Oxford Open Neuroscience or any OUP journal. Please note that OUP will only ever request payment for applicable fees be made via SciPris or to an OUP bank account. If you ever have concerns about the legitimacy of a request, please do not hesitate to contact a customer services agent via the SciPris portal or directly via oupsupport@scipris.com.

Thank you for your fine contribution. On behalf of the Editors of the Oxford Open Neuroscience, we look forward to your continued contributions to the Journal.

Sincerely,

Dr. Carlos Ibanez

Senior Editor, Oxford Open Neuroscience

carlos.ibanez@pku.edu.cn

Date Sent:

17-Mar-2023
